# *Ochrobactrum* sp. MPV1 from a dump of roasted pyrites can be exploited as bacterial catalyst for the biogenesis of selenium and tellurium nanoparticles

**DOI:** 10.1186/s12934-017-0826-2

**Published:** 2017-11-28

**Authors:** Emanuele Zonaro, Elena Piacenza, Alessandro Presentato, Francesca Monti, Rossana Dell’Anna, Silvia Lampis, Giovanni Vallini

**Affiliations:** 1 Department of Biotechnology, University of Verona, Strada le Grazie 15, 37134 Verona, Italy; 2 Microbial Biochemistry Laboratory, Department of Biological Sciences, University of Calgary, 2500 University Dr. NW, Calgary, AB T2N 1N4 Canada; 3 Department of Computer Science, University of Verona, Strada Le Grazie 15, 37134 Verona, Italy; 4 Micro Nano Facility, Fondazione Bruno Kessler, Via Sommarive 18, 38123 Povo (TN), Italy

**Keywords:** Aerobic selenite reduction, Aerobic tellurite reduction, Bacterial-metalloid interactions, Biogenically synthesized nanoparticles, Chalcogen metalloids, *Ochrobactrum* sp. MPV1, Rare earth oxyanions

## Abstract

**Background:**

Bacteria have developed different mechanisms for the transformation of metalloid oxyanions to non-toxic chemical forms. A number of bacterial isolates so far obtained in axenic culture has shown the ability to bioreduce selenite and tellurite to the elemental state in different conditions along with the formation of nanoparticles—both inside and outside the cells—characterized by a variety of morphological features. This reductive process can be considered of major importance for two reasons: firstly, toxic and soluble (i.e. bioavailable) compounds such as selenite and tellurite are converted to a less toxic chemical forms (i.e. zero valent state); secondly, chalcogen nanoparticles have attracted great interest due to their photoelectric and semiconducting properties. In addition, their exploitation as antimicrobial agents is currently becoming an area of intensive research in medical sciences.

**Results:**

In the present study, the bacterial strain *Ochrobactrum* sp. MPV1, isolated from a dump of roasted arsenopyrites as residues of a formerly sulfuric acid production near Scarlino (Tuscany, Italy) was analyzed for its capability of efficaciously bioreducing the chalcogen oxyanions selenite (SeO_3_^2−^) and tellurite (TeO_3_^2−^) to their respective elemental forms (Se^0^ and Te^0^) in aerobic conditions, with generation of Se- and Te-nanoparticles (Se- and TeNPs). The isolate could bioconvert 2 mM SeO_3_^2−^ and 0.5 mM TeO_3_^2−^ to the corresponding Se^0^ and Te^0^ in 48 and 120 h, respectively. The intracellular accumulation of nanomaterials was demonstrated through electron microscopy. Moreover, several analyses were performed to shed light on the mechanisms involved in SeO_3_^2−^ and TeO_3_^2−^ bioreduction to their elemental states. Results obtained suggested that these oxyanions are bioconverted through two different mechanisms in *Ochrobactrum* sp. MPV1. Glutathione (GSH) seemed to play a key role in SeO_3_^2−^ bioreduction, while TeO_3_^2−^ bioconversion could be ascribed to the catalytic activity of intracellular NADH-dependent oxidoreductases. The organic coating surrounding biogenic Se- and TeNPs was also characterized through Fourier-transform infrared spectroscopy. This analysis revealed interesting differences among the NPs produced by *Ochrobactrum* sp. MPV1 and suggested a possible different role of phospholipids and proteins in both biosynthesis and stabilization of such chalcogen-NPs.

**Conclusions:**

In conclusion, *Ochrobactrum* sp. MPV1 has demonstrated to be an ideal candidate for the bioconversion of toxic oxyanions such as selenite and tellurite to their respective elemental forms, producing intracellular Se- and TeNPs possibly exploitable in biomedical and industrial applications.
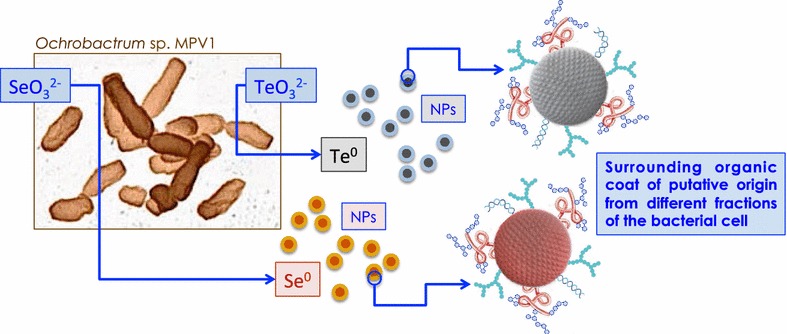

## Background

The strain *Ochrobactrum* sp. MPV1, a strict aerobic α-proteobacterium, object of the present study was isolated from a dump of roasted pyrite at the Nuova Solmine SpA industrial site near Scarlino (Loc. Il Casone, 42°92′56.58″N|10°79′61.7″E) in Southern Tuscany, Italy [1, 2]. This area has been dedicated for decades to the production of sulfuric acid by roasting pyrites (including mainly pyrite sensu stricto, chalcopyrite and arsenopyrite) from the mines of the neighbouring Colline Metallifere (Metalliferous Hills), during the period from the beginning of the twentieth century to the definitive closure of the mining activities in 1996. Roasted pyrite residues are reported to be severely contaminated by a variety of heavy metals such as Co, Cr, Cu, Fe, Mn, Pb and Zn as well as arsenic (As) [3], but even moderate levels of Ba, Ni, Se and V can be found associated to them [4]. Therefore, bacterial strains harboured in matrices like these have necessarily been selected to face high concentrations of toxic elements.

In particular, Se—along with the other chalcogen tellurium (Te)—is a widely spread environmental contaminant. Both these metalloids enter different habitats either from natural sources or because of anthropic activities [5]. They share several physico-chemical properties. Se and Te occur in nature in four valence states, namely + 6, + 4, 0 and − 2, and form oxyanions, selenite and selenate (SeO_3_^2−^, SeO_4_^2−^) on one hand and tellurite and tellurate (TeO_3_^2−^, TeO_4_^2−^) on the other, that are highly toxic to many living beings from bacteria to mammals [6, 7]. Interestingly, *Ochrobactrum* sp. MPV1, grown in the presence of selenite and tellurite, evidenced high tolerance to both oxyanions as well as the capability of aerobically reducing SeO_3_^2−^ and TeO_3_^2−^ to zero-valent elemental Se^0^ and Te^0^, in form of colloidal nanoparticles (NPs). Indeed, the reduction of selenium and tellurium oxyanions under aerobic conditions is well documented for axenic cultures of a variety of bacterial strains [8–11].

From an environmental point of view, it is worth noting that—as mentioned before—the tellurium oxyanion represents a chemical species highly toxic to the biota [12], including most bacteria, against which toxicity occurs at concentrations as low as 1 µg mL^−1^ [13]. This trait is even more impressive when a comparison is made with the other chalcogen oxyanion SeO_3_^2−^, which—although toxic as well—exerts toxicity at concentrations about 100-fold higher than that of TeO_3_^2−^ [14]. The toxic character of these compounds has been related to their oxidizing capacity leading to the production of reactive oxygens species (ROS) as a consequence of the interaction with the cellular thiols: redox buffering system [15].

Not to mention the role that selenite- and tellurite-reducing bacteria such as *Ochrobactrum* sp. MPV1 can take on in terms of abatement of these toxic oxyanions from environmental matrices (i.e. soils, sediments, aquifers, and wastewater streams), it is their ability to produce elemental Se and Te nanoparticles that is assuming increasing relevance due to the potential use of these NPs based on their unique physical and chemical properties as well as a pronounced biological reactivity. Actually, these chalcogen NPs have stimulated growing interests particularly because of their photoelectric and semiconducting characteristics. For instance, SeNPs have been proposed as enhancing materials for H_2_O_2_ biosensors [16]. On the other hand, TeNPs have been envisaged for the production of optoelectronics devices [17]. Moreover, the exploitation of these nanoparticles as antimicrobial agents alternative to the traditional antibiotics is currently becoming an area of intensive research in the medical and health-care sector [18–21].

A last aspect should be considered. Obtaining metal/metalloid NPs using biological systems represents a valuable alternative to the chemical synthesis since biogenic production occurs under mild physico-chemical conditions allowing to lower costs for required reagents and energy as well as to reduce generation of hazardous residues [22]. Furthermore, biogenic selenium and tellurium NPs are surrounded by organic layers which include proteins, lipids and carbohydrates [23–25]. These organic coats greatly influence the stability of such NPs of biogenic origin and therefore their reactivity [26]. Buchs et al. [27] have already deeply investigated the colloidal stability of biogenic SeNP suspensions. It was stated that biogenic SeNPs are colloidally stable at physiological pH values, above 5.4, due to their intrinsic negative charge (<− 30 mV). More recently, Mal et al. [28] by comparing the toxicity of biogenic SeNPs versus chemically synthesized ones towards Zebrafisch embryos suggested that the organic layer surrounding biogenic SeNPs is constituted of components of the extracellular polymeric matrix, which govern the physiochemical stability and surface properties.

Nevertheless, the precise biochemical mechanisms involved in the reduction of SeO_3_^2−^ and TeO_3_^2−^ oxyanions to their elemental forms are still matter of debate and waiting to be fully understood.

The study here presented reports on the bioprecipitation ability of *Ochrobactrum* sp. MPV1 toward selenite and tellurite, with intracellular accumulation of Se^0^ or Te^0^ nanoparticles as ascertained through SEM–EDX and TEM analyses. Additional analytical approaches were performed in the attempt to shed light on the mechanisms involved in selenite and tellurite reduction to their respective elemental forms as well as to characterize the NPs generated.

Evidences achieved in the present study suggest that *Ochrobactrum* sp. MPV1 can become a candidate as biocatalyst for the synthesis of Se^0^ and Te^0^ nanoparticles in view of their possible technological exploitation.

## Methods

### Chemicals, culture media and solutions

Chemicals purchased from Sigma-Aldrich^®^ (Milan, Italy) were all analytical grade. Nutrient broth, and Bacteriological Agar were furnished by Oxoid Italia Spa (Garbagnate Milanese, Italy). Defined medium (DM) was prepared as described by Frassinetti et al. [29]. Na_2_SeO_3_ and K_2_TeO_3_ were prepared as a 100 mM stock solution in deionized water and sterilized by filtration.

### Bacterial identification

The bacterial strain MPV1 has been identified within the microbial community harbored in a sample of roasted arsenopyrite collected at the formerly dumping site for such an industrial waste near the facilities of a factory (Nuova Solmine SpA, Tuscany, GR, Italy), which has been historically operating for the production of sulfuric acid by using pyrites as starting raw material [1]. Genomic DNA was extracted and purified from 18-h culture of MPV1 strain grown on nutrient broth using chloroform–phenol method. Universal 16S rRNA PCR primers F8 and R11 [30] were used in the amplification of 16S rRNA gene. Conditions for gene amplification were as follow: an initial denaturation temperature of 95 °C for 5 min, a run of 30 cycles with each cycle consisting of 1 min at 95 °C, 1 min at 50 °C and 2 min at 72 °C and a final extension step at 72 °C for 5 min.

PCR products were cloned into pGEM-T vector using Easy T-Vector System (Promega, Italy) followed by sequencing of both strands (Primm, Italy). The sequence was searched for homology using BLASTN database [31] and EZ Taxon-E database [32].

Multiple nucleotide sequences alignments were constructed using CLUSTAL_W 1.83 [33]. A phylogenetic tree was then constructed based on the neighbor-joining method using the MEGA version 6.0 software package [34] with 1000 data sets examined by bootstrapping.

### Determination of SeO_3_^2−^ and TeO_3_^2−^ minimum inhibitory concentration (MIC)

In order to establish the MIC of either SeO_3_^2−^ (MIC^Se^) or TeO_3_^2−^ (MIC^Te^), *Ochrobactrum* sp. MPV1 was pre-cultured in a test tube (Sarstedt) containing 5-mL of nutrient broth medium (here indicated as NB) until the stationary growth phase (18-h) at 27 °C with shacking (200 rpm). MPV1 cells were then challenged in a test tube containing 5-mL of fresh NB medium amended with increasing concentrations of either SeO_3_^2−^ (0–100 mM) or TeO_3_^2−^ (0–3 mM). After 24-h exposure to chalcogen-oxyanions, an aliquot of MPV1 cells were spotted onto NB agar plates and recovered for further 24-h at 27 °C to establish the concentration of either SeO_3_^2−^ or TeO_3_^2−^ inhibiting the bacterial growth.

### Evaluation of bacterial growth dynamic after exposure to SeO_3_^2−^ or TeO_3_^2−^

MPV1 growth dynamic was determined inoculating 250-mL Erlenmeyer flasks containing 100 mL of NB medium supplied either with Na_2_SeO_3_ (0.5 or 2 mM) or K_2_TeO_3_ (0.3, 0.5 or 1 mM). Thus, 100 μL of MPV1 cells were sampled every 24-h from each culture, being then serially diluted in sterile saline solution (NaCl 0.9% w/v) and seeded onto NB agar recovery plates, which were incubated at 27 °C for 24-h. The number of growing cells is reported as average (n = 3) of the Colony Forming Unit per milliliter (CFU/mL) with standard deviation.

### Evaluation of selenite and/or tellurite bioreduction efficiency

MPV1’s efficiency in reducing SeO_3_^2−^ and/or producing Se^0^ was measured spectrophotometrically by using the method described by Kessi et al. [35] and Biswas et al. [36] respectively, while its ability to remove TeO_3_^2−^ was assessed following the method published by Turner et al. [37], over the incubation timeframe tested. Finally, inductively coupled plasma mass spectrometry (ICP-MS) was performed on Te-nanostructures recovered from 0.3 mM TeO_3_^2−^-grown cells for 0, 24, 48 and 96-h to establish the extent of Te^0^ produced by MPV1 strain.

### Effect of MPV1 pre-induction towards selenite and/or tellurite bioreduction

MPV1 cells pre-induced for 24 h with sub-lethal concentrations of selenite (0.3 mM) or tellurite (0.1 mM) were subsequently grown in the presence of either SeO_3_^2−^ (2 mM) or TeO_3_^2−^ (0.5 mM), in order to assess the bioreduction capability of chalcogen-oxyanion adapted biomasses. The residual SeO_3_^2−^ and TeO_3_^2−^ concentrations in the medium were measured following the above described methods [35, 37].

### Separation of the subcellular fractions

Different subcellular fractions (cytoplasm, periplasm and membranes) were tested for SeO_3_^2−^ and TeO_3_^2−^ reduction activities. Bacterial cells were recovered, centrifuged and washed twice with 400 mL of saline solution. Afterwards, cells were subjected to periplasmic solubilization according to the method of Osborn and Munson [38]. Spheroplasts were harvested by centrifugation at 25,000×*g* for 20 min and re-suspended in 10 mL of a solution containing 50 mM NaCl and one tablet of cOmplete™, Mini EDTA-free Protease Inhibitor, while the supernatant containing the periplasmic fraction was recovered, filtered (0.2-µm filter) and stored at − 20 °C. Spheroplasts were then disrupted by sonication and the solution was centrifuged at 200,000×*g* for 75 min. After centrifugation, the soluble cytoplasmic fraction present in the supernatant was recovered, filtered and stored at − 20 °C.

On the other hand, the membrane fraction, visible as a brown pellet, was solubilized in 10 mL of a 50 mM Phosphate Buffered Saline (PBS; 11.2 mM KH_2_PO_4_, 38.8 mM K_2_HPO_4_, pH 7.4) containing 0.5% v/v Triton X-100, frozen and stored at − 20 °C.

### In vitro SeO_3_^2−^ and TeO_3_^2−^ reduction assays

The reduction activities of proteins contained in the recovered subcellular fractions towards SeO_3_^2−^ and TeO_3_^2−^ were evaluated by using a 96 well microtiter plate where 50 µL of protein sample (100 ng of proteins), 148 µL of McIlvaine buffer at different pH values (6.0, 6.5, 7.0), 2 µL of NADH (final concentration 2.0 mM) and 10 µL of Na_2_SeO_3_ or K_2_TeO_3_ (final concentration 5 mM) were added in each well. The mixture was then incubated at room temperature for 24-h and the production of Se^0^ and Te^0^ was observed through the development of red or black colors within the wells. Additionally, the cytoplasmic fraction activity towards either SeO_3_^2−^ or TeO_3_^2−^ in vitro reduction was also spectrophotometrically evaluated in the presence of 2.0 mM of NADH, NADPH and reduced ascorbate as electron donors, by reading the absorbance values of the elemental forms produced at 415 nm (Se^0^) and 595 nm (Te^0^) wavelength.

### Measurement of NAD^+^/NADH ratio

NAD^+^/NADH ratio of a solution containing 50 µL of cytoplasmic fraction recovered from MPV1 cells, 148 µL of McIlvaine buffer at pH 6.5, 2 µL of NADH (final concentration 2.0 mM) and 10 µL of Na_2_SeO_3_ or K_2_TeO_3_ (final concentration 5 mM) was quantified in vitro using an enzyme cycling assay, which was performed at room temperature and adapted for measurement in a microtiter plate [39]. Briefly, 5 µL of the above mixture were taken every 12-h and added to 90 μL aliquots of a master reagent mix containing bicine buffer (1 M, pH 8), 40 mM EDTA, 4.2 mM thiazolyl blue and 6.6 mM phenazine ethosulfate, which was previously warmed to 30 °C. The reaction mixture was incubated for 10 min at 30 °C, and then the cycling reaction was started by the addition of 5 μL 0.1 M bicine (pH 8.0) containing 347 units mL^−1^ of alcohol dehydrogenase (Sigma-Aldrich^®^). The absorbance of the reaction mixture was read at 570 nm to measure the NAD^+^/NADH ratio referring to a calibration curve obtained using standards solutions with known ratios of NAD^+^/NADH (Sigma-Aldrich^®^). The data are reported as average of a biological triplicate (n = 3) with standard deviation.

### BSO inhibition test

*Ochrobactrum* sp. MPV1 was inoculated in 30 mL of NB medium supplemented with 2 mM Na_2_SeO_3_ or 0.3 mM K_2_TeO_3_ and with two different concentrations (1 mM, 3 mM) of *S*-*n*-butyl homocysteine sulfoximine (BSO), which is an inhibitor of glutathione (GSH) biosynthesis [40]. Thus, BSO effect upon MPV1 cells bioreduction of either SeO_3_^2−^ or TeO_3_^2−^ over the time was evaluated by monitoring their removal, as published elsewhere [35, 37].

### GSH activity towards SeO_3_^2−^ and/or TeO_3_^2−^ and measurement of RSH content

The potential involvement of RSH-containing molecules in SeO_3_^2−^ and/or TeO_3_^2−^ reduction was assessed comparing in vitro the reduction activity of MPV1 cytoplasmic fraction with physiological concentrations of L-GSH (5 and 10 mM) [41] towards 0.5 mM SeO_3_^2−^ or 0.3 mM TeO_3_^2−^, Experiments were carried out under room temperature by using 100 ng of cytoplasmic proteins per reaction. The absorbance values of the Se^0^ and Te^0^ were read at 415 and 595 nm, respectively.

RSH content of MPV1 cells grown in the presence of chalcogen-oxyanions was measured as described by Turner et al. [42]. Briefly, 1 mL of either 0.5 mM SeO_3_^2−^- or 0.3 mM TeO_3_^2−^-grown MPV1 cells were collected at different time points (0, 12, 24, 36, 48, 60 and 72-h) and centrifuged for 10 min at 15,000×g. Bacterial cell pellets were then re-suspended in 1 mL of Ellman’s reagent containing 50 mM Tris HCl pH 8.0, 5 mM EDTA, 0.1% SDS and 0.1 mM 5,5′-dithiobis(2-nitrobenzoic acid) (DTNB). Finally, the suspensions were incubated at 37 °C for 30 min, being then centrifuged for 10 min at 15,000×g and the absorbance of the supernatant read at 412 nm.

### Scanning electron microscopy

*Ochrobactrum* sp. MPV1 was exposed to either Na_2_SeO_3_ (2 mM) or K_2_TeO_3_ (0.5 mM) for 24-h, being then the cells harvested by centrifugation (6000×*g* for 10 min) and washed three times with PBS (4.3 mM KH_2_PO_4_, 1.47 mM K_2_HPO_4_ pH 7.4) prior their fixation with a 2.5% v/v glutaraldehyde solution in 0.1 M PBS. Once fixed, the cells were dehydrated with increasing ethanol concentrations (from 30 to 100%), mounted onto metallic stubs and sputter-coated with carbon (MED 010 Balzers). SEM observations were performed using the back-scattered electron (BSE) emission mode with XL30 ESEM (FEI-Philips) equipped with an EDAX (FEI-Philips) micro-analytical system.

### Transmission electron microscopy

The MPV1 strain was inoculated in NB medium containing either 2 mM of Na_2_SeO_3_ or 0.5 mM of K_2_TeO_3_ and bacterial cells were collected after 24, 48 and 72-h of growth, being subsequently harvested through centrifugation (6000*g* × 10 min). Then, MPV1 cells were firstly fixed with a solution containing paraformaldehyde (4% w/v; EM grade) and glutaraldehyde (2% w/v; EM grade, TAAB, England) in cacodylic buffer (0.1 M pH 7.2) for 30 min, and subsequently incubated for further 30 min in the same buffer containing paraformaldehyde (4% w/v; EM grade), being then spinned down using a bench centrifuge at 6000×*g* for 10–20 min and washed three times in cacodylic buffer. Afterwards, bacterial cells were incubated in osmium tetroxide (1% v/v) (TAAB) dissolved in cacodylic buffer for 1-h in the dark to counter-fix the membranes. Thus, three washing steps in distilled water (10 min each) were performed, and the samples were subsequently incubated for 1-h in a solution containing uranyl acetate (1% w/v) (SIGMA, England) in water [40]. After washes with distilled water, MPV1 cells were dehydrated in water/ethanol solutions (50, 70, 90, and 100%). A double wash in propylene oxide for 10 min was performed, followed by incubation of the samples in propylene oxide: TAAB LV Resin (TAAB, England) 2:1, 1:1, 1:2 for 1-h each, and then in pure resin TAAB LV Resin for a further 1-h. The cells with fresh resin were incubated for 24-h in an oven at 65 °C. Once the cells were included into the resin blocks, thin sections (70 nm) were cut with PowerTome Ultramicrotome (RMC, UK) and collected onto Formvar-coated copper slot grids, which were post-stained with aqueous uranyl acetate or Reynold’s lead citrate (EMS) to enhance contrast/visualization. Thin sections were imaged using a JEOL 1014 electron microscope (JEOL, Japan) operated at 80 kV to assess the quality of ultrastructural preservation, collecting sets of 2D images.

### Extraction of biogenic SeNPs and TeNPs

Se and TeNPs were recovered from *Ochrobactrum* sp. MPV1 cultures after 24 and 48-h growth on NB medium supplied with 2.0 Na_2_SeO_3_ and 0.3 mM K_2_TeO_3_, respectively. Bacterial cells and NPs were collected by centrifuging at 10,000×*g* for 10 min. The pellets were washed twice with saline solution, re-suspended in Tris/HCl 1.5 M buffer (pH 8.2) and the cells were then disrupted by ultrasonication at 100 W for 5 min. The suspensions were centrifuged at 10,000×*g* for 30 min to separate the cellular debris (pellet) from NPs (supernatant). NPs were recovered after centrifugation at 40,000×*g* for 30 min, washed twice with water and re-suspended in deionized water [20].

### FT-IR (Fourier transform infrared spectroscopy) and PCA (principal component analysis)

5 µL aliquots of SeNPs and TeNPS collected after 24 (T1) and 48-h (T2) of incubation were settled down onto BaF_2_ supports and dried for 24-h at 40 °C before measurement.

Mid-infrared spectra were acquired in transmission mode in the 4000–700 cm^−1^ range using a Vertex 70 Bruker spectrometer coupled to a Hyperion 3000 vis/IR microscope equipped with a photoconductive MCT detector and a 15 × objective. For all the samples, at least 8 point by point spectra were acquired at 4 cm^−1^ resolution on a 50 µm × 50 µm area by co-adding 64 scans (about 30 s acquisition time). Absorption spectra were baseline corrected with the rubberband method and area-normalized in the 4000–2400 cm^−1^ range and in the 1800–700 cm^−1^ range separately.

For a better identification of most significant differences in the biochemical composition of Se and TeNPs at the two incubation times, principal component analysis (PCA) was carried out on the baseline corrected and area normalized FT-IR spectra after mean-centering each spectral channel across all the measurements, using the statistical package ChemoSpec developed in the R software environment [43].

For a given set of spectra, PCA allows the representation of each spectrum of the data set through its components (scores) onto so called ‘principal components’ (PCs). PCs are calculated and ordered in such a way that the first principal component PC1 accounts for the maximum variance in the original data set; the second principal component PC2 is orthogonal (uncorrelated) to the first one and accounts for most of the remaining variance and so on. For each PC, the components on each spectral channel are called ‘loadings’.

## Results

### Sequence analysis of the 16S rRNA gene

**Fig. 1:**
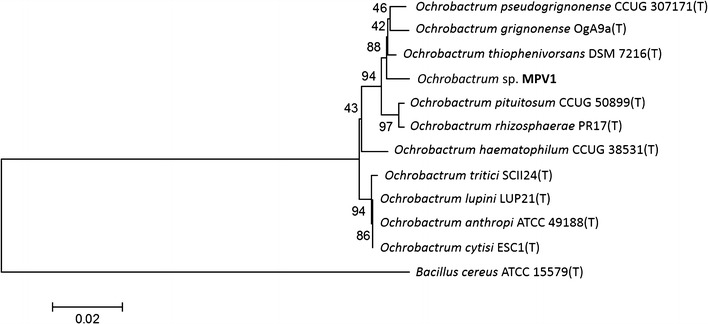
Neighbor-joining phylogenetic tree based on the sequence of 16s rRNA gene showing the phylogenetic position of strain MPV1. Bootstrap values are shown for nodes that had > 50% support in a bootstrap analysis of 1000 replicates. The scale bars indicate the number of substitutions per nucleotide position

A 1523-bp fragment of 16S rRNA gene from the MPV1 strain was sequenced and identity values were obtained through EZ-Taxon server. The isolate showed an identity percentage of 99.13% with *Ochrobactrum thiophenivorans*, 98.99% with *Ochrobactrum pseudogrignonense* and 98.89% with *Ochrobactrum grignonense*. In the neighbour joining (N-J) phylogenetic tree (Fig. 1) the strain is placed next to *O. thiophenivorans*. It was tentatively identified as an *Ochrobactrum* sp.

### MPV1’s tolerance towards chalcogen-oxyanions

**Fig. 2:**
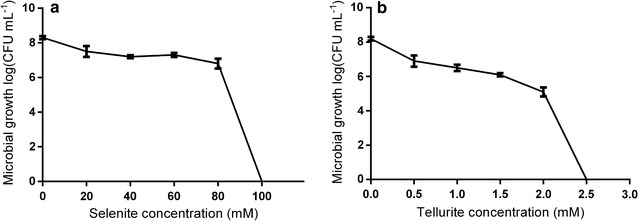
Kill curve of *Ochrobactrum* sp. MPV1. The strain was exposed to increasing concentrations of SeO_3_^2−^ (**a**) and TeO_3_^2−^ (**b**)

The tolerance of *Ochrobactrum* sp. MPV1 towards metalloid-oxyanions was evaluated by challenging the isolate with increasing concentrations of SeO_3_^2−^ and TeO_3_^2−^. The MPV1 strain was able to survive up to 80 mM Na_2_SeO_3_ (MIC^Se^) and 2.0 mM K_2_TeO_3_ (MIC^Te^), whose toxicity was already (Fig. 2) noticeable at a concentration of 1.5 mM K_2_TeO_3_, with a reduction of ca. 2 Log unit (Fig. 2b).

### MPV1 bioreduction of SeO_3_^2−^ and TeO_3_^2−^ and cellular pre-induction effect

**Fig. 3:**
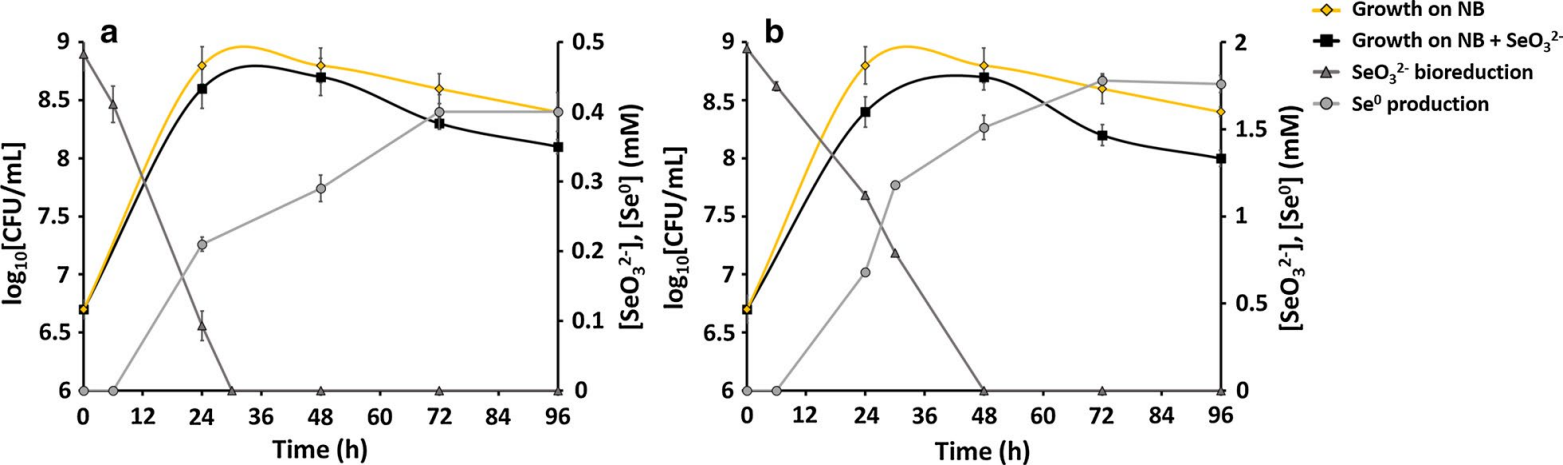
Time courses of bacterial growth, SeO_3_^2−^ depletion and Se^0^ formation by *Ochrobactrum* sp. strain MPV1. Tests were carried in presence of **a** 0.5 and **b** 2.0 mM SeO_3_^2−^. Each curve shows means based on the results of three experiments

MPV1 growth was slightly affected by the presence of the two different SeO_3_^2−^ concentrations (0.5 and 2 mM) tested as compared to the control growth profile (absence of SeO_3_^2−^) (Fig. 3). The cultures turned red as growth progressed, suggesting SeO_3_^2−^ bioconversion to the red form of Se^0^. Indeed, the biotic reduction of both 0.5 and 2 mM SeO_3_^2−^ was completed within 30 and 48-h of the bacterial growth, respectively (Fig. 3a, b). Consequently, Se^0^ production by the isolate started after 6-h of incubation for both SeO_3_^2−^ concentrations tested. Aside from the initial delay, Se^0^ production was almost concurrent with SeO_3_^2−^ depletion. After 96-h of incubation, ~ 92% of Na_2_SeO_3_ was converted into Se^0^.

**Fig. 4:**
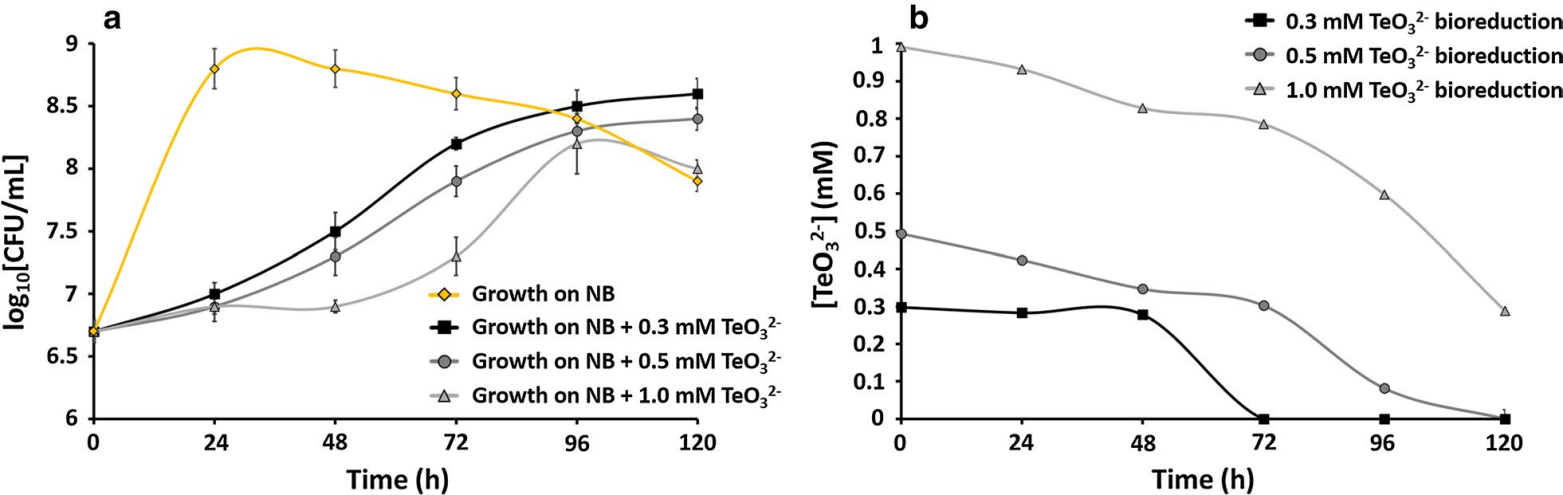
Time course of bacterial growth (**a**) and TeO_3_^2−^ reduction (**b**) by *Ochrobactrum* sp. strain MPV1. Tests were carried in presence of 0.3, 0.5 and 1 mM TeO_3_^2−^. Each curve shows means based on the results of three experiments

On the other hand, we detected a noticeable negative effect on the growth of the MPV1 strain exposed to all three TeO_3_^2−^ concentrations examined (0.3, 0.5 and 1 mM), which was underlined by the delayed cellular growth during the earliest and mid log phases (Fig. 4a). In addition, the highest TeO_3_^2−^ concentration (1 mM) tested showed a far more striking negative effect upon the bacterial growth. TeO_3_^2−^ bioreduction led to the typical blackening of MPV1’s cultures after 48–72-h of incubation, suggesting the accumulation of Te^0^. Particularly, the MPV1 strain completely removed both 0.3 and 0.5 mM TeO_3_^2−^ in 72 and 120-h respectively, while ca. 70% of 1 mM TeO_3_^2−^ was bioreduced over the timeframe considered (Fig. 4b). As a consequence of TeO_3_^2−^ bioreduction, the amount of Te^0^ bioproduced in the form of nanostructures resulted to increase over the incubation time, being 0.029 ± 0.001, 0.069 ± 0.001 and 0.263 ± 0.022 mM after 24, 48 and 96-h, respectively.

**Fig. 5:**
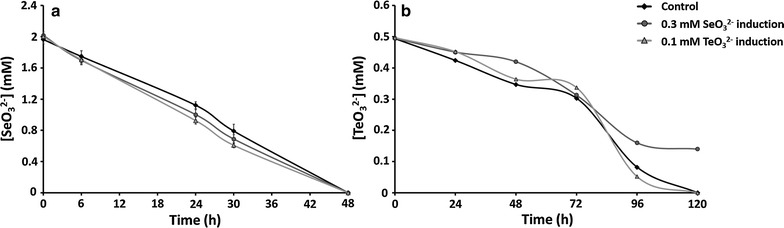
Effect of pre-induction on SeO_3_^2−^ (**a**) and TeO_3_^2−^ (**b**) reduction in *Ochrobactrum* sp. MPV1. Pre-inductions were carried with sub-lethal concentration of selenite (0.3 mM) and tellurite (0.1 mM)

The induction of the MPV1 strain by exposing the bacterial cells for 24-h either to 0.3 mM SeO_3_^2−^ or 0.1 mM TeO_3_^2−^ and subsequently incubating them in the presence of 2 mM SeO_3_^2−^ did not revealed over the time a direct effect on the bioreduction of both chalcogen-oxyanions (Fig. 5a). Although a similar behavior was observed for the MPV1 strain pre-induced on 0.1 mM TeO_3_^2−^ and then exposed to 0.5 mM TeO_3_^2−^, the induction of bacterial cells on 0.3 mM SeO_3_^2−^ led instead to a decrease of TeO_3_^2−^ bioreduction extent, being even lower than the one of not induced cells (Fig. 5b). Thus, these results suggested the existence of different bioprocessing mechanisms of both SeO_3_^2−^ and TeO_3_^2−^ exploited by the MPV1 strain.

### Localization of SeNPs and Te-nanostructures in *Ochrobactrum* sp. MPV1 cells

**Fig. 6:**
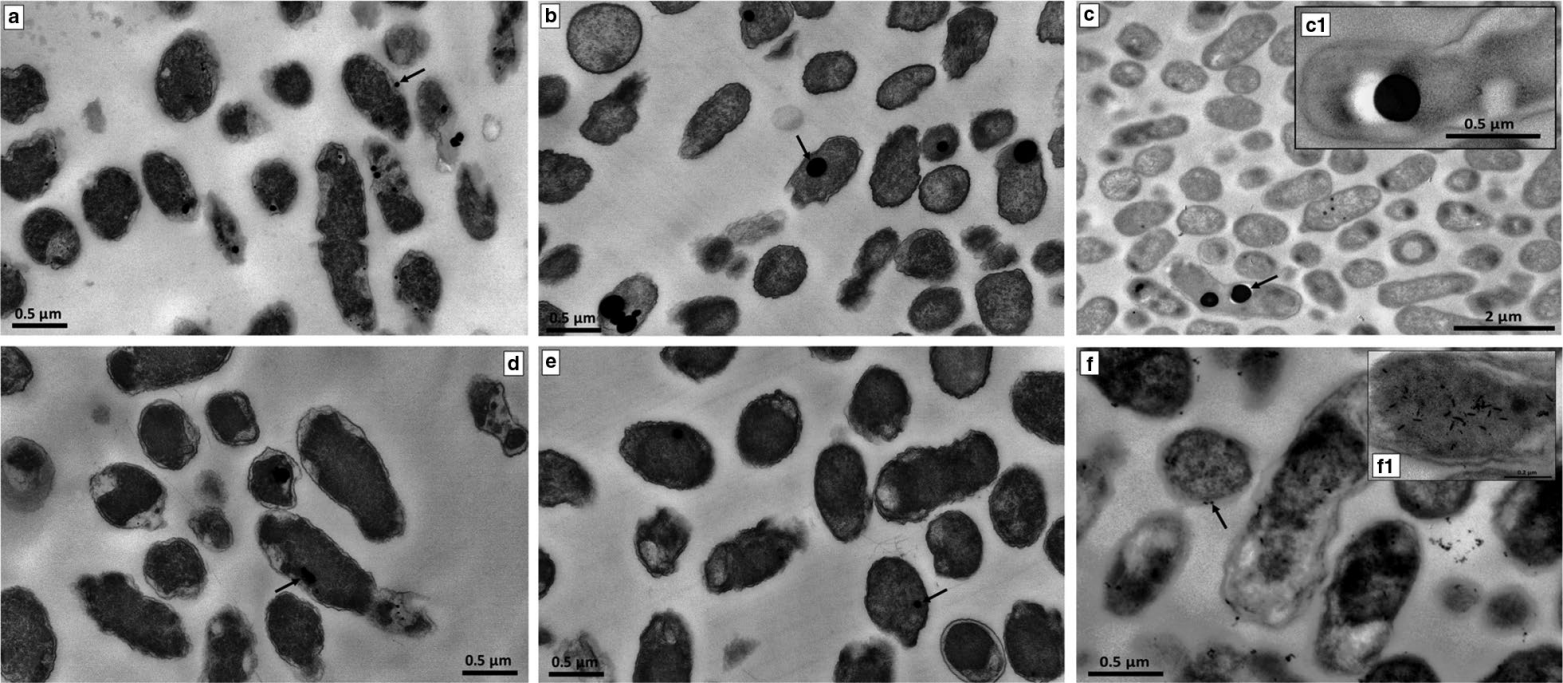
TEM micrographs of *Ochrobactrum* sp. MPV1 exposed to SeO_3_^2−^ and TeO_3_^2−^. Cultures were grown in presence of 2 mM SeO_3_^2−^ for 24 (**a**), 48 (**b**) and 72-h (**c**, **c1**) or 0.3 mM TeO_3_^2−^ for 24 (**d**), 48 (**e**) and 72-h (**f**, **f1**)

TEM observations revealed the production of intracellular electron-dense NPs after 24 and 48-h of MPV1 growth in the presence of both 2 mM SeO_3_^2−^ (Fig. 6a, b) and 0.5 mM TeO_3_^2−^ (Fig. 6d, e), as indicated by black arrows. After 72-h of MPV1 exposure to oxyanions, NPs were detected only in the case of SeO_3_^2−^-grown cells (Fig. 6c, c1), while those grown in the presence of TeO_3_^2−^ displayed intracellular electron-dense focuses resembling short needle-like Te-nanorods (TeNRs) other than spherical particles (Fig. 6f, f1). In this regard, SeNPs size increases over the timeframe considered, as shown by the formation of bigger NPs within SeO_3_^2−^-grown cells as compared to the particles observed at 24 and 48-h growth (Fig. 6a–c). On the other hand, TeO_3_^2−^-exposed cells tuned the Te-nanostructure morphology from NPs to short NRs (Fig. 6d–f).

**Fig. 7:**
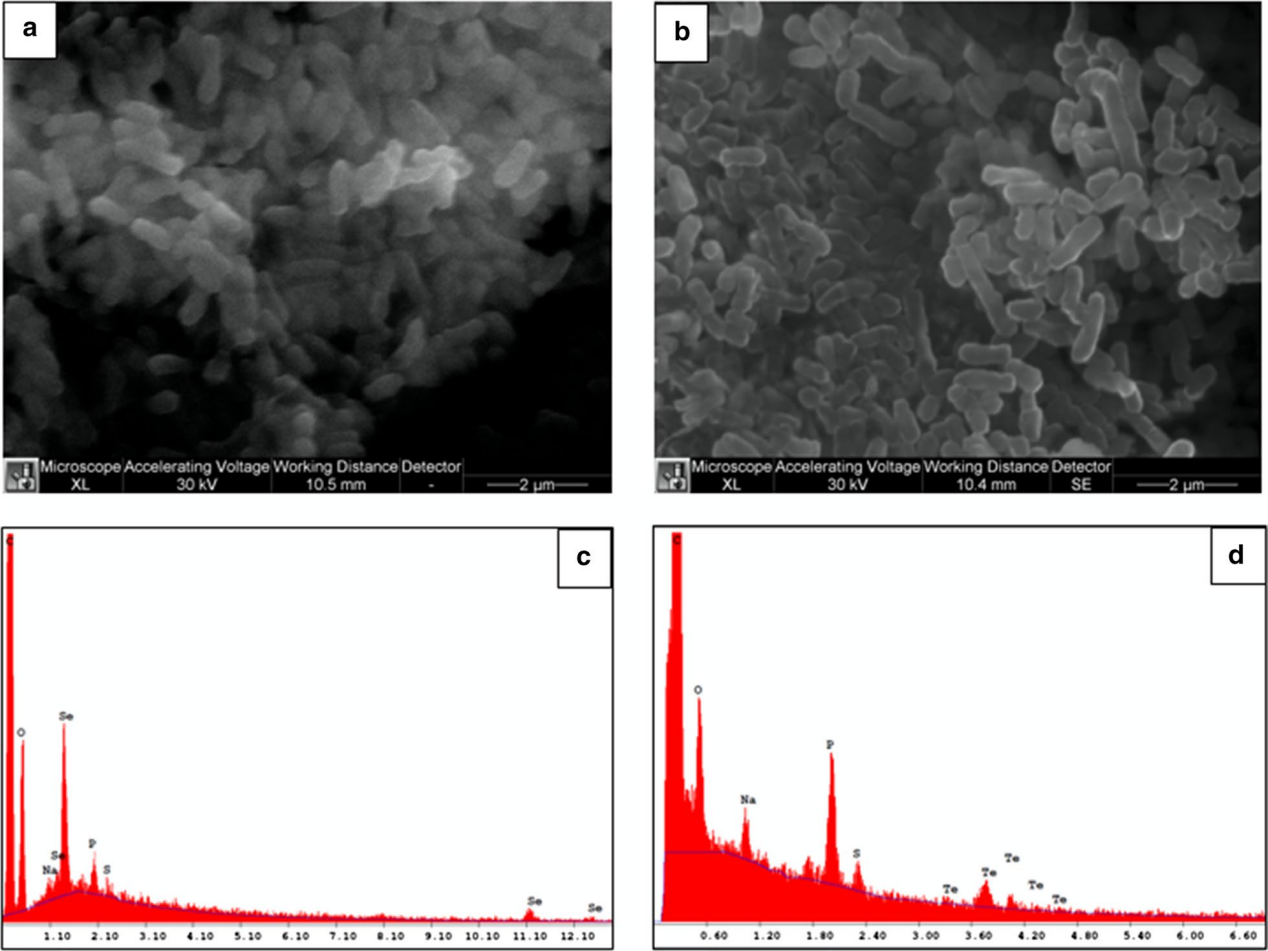
SEM micrographs (**a**, **b**) and EDAX spectra (**c**, **d**) of *Ochrobactrum* sp. MPV1. Culture were grown in presence of 2.0 mM SeO_3_^2−^ (**a**) or 0.3 mM TeO_3_^2−^ (**b**)

The intracellular production of Se- and Te-nanomaterials was confirmed by SEM micrographs of bacterial cells grown in the presence of either SeO_3_^2−^ or TeO_3_^2−^, in which no extracellular nanosized material was detected (Fig. 7a, b). EDX spectra confirmed the intracellular localization of SeO_3_^2−^ and TeO_3_^2−^, revealing the specific selenium absorption peaks at 1.37, 11.22, 12.49 keV (Fig. 7c), and K-alpha absorption peak of tellurium at 3.769 keV (Fig. 7d).

### Interpretation of putative SeO_3_^2−^ or TeO_3_^2−^ reduction mechanisms

**Fig. 8:**
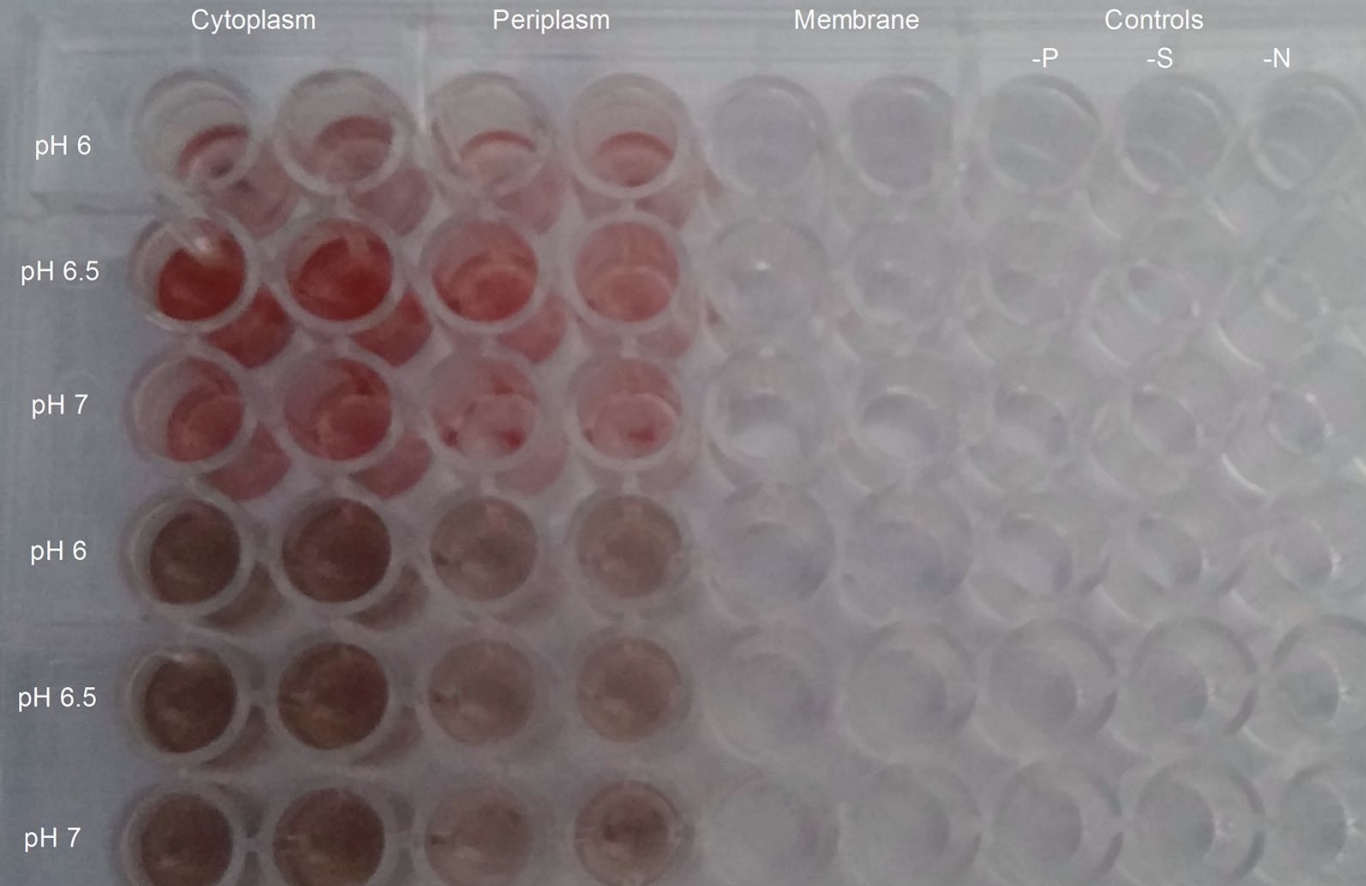
SeO_3_^2−^ and TeO_3_^2−^ reduction activity test. Different subcellular fractions (cytoplasm, periplasm, membrane) were tested for SeO_3_^2−^ and TeO_3_^2−^ reduction at different pH values in the presence of NADH as electron donor

**Fig. 9:**
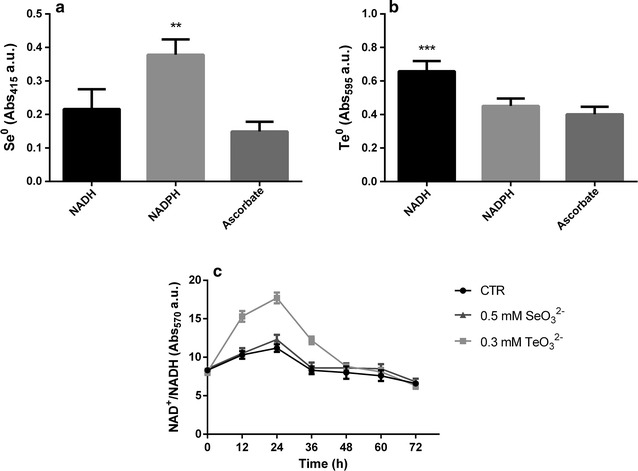
Effect of different electron donors on SeO_3_^2−^ (**a**) and TeO_3_^2−^ (**b**) reduction and measurement of NAD^+^/NADH ratio (**c**). Three different electron donors were evaluated in **a**, **b**: NADH, NADPH, ascorbate

Different cell fractions (i.e. cytoplasmic, periplasmic and membrane-associated) buffered at diverse pH values (6.0, 6.5, 7.0) and supernatant from liquid cultures were tested for SeO_3_^2−^ or TeO_3_^2−^ reduction activities. As shown in Fig. 8, in vitro reduction of both SeO_3_^2−^ and TeO_3_^2−^ occurred in the cytoplasmic and periplasmic fractions at different pH values, upon addition of NADH as electron donor. Further, the formation of Se^0^ and Te^0^ by the cytoplasmic fraction recovered from MPV1 cells revealed that NADPH exhibited the highest SeO_3_^2−^ reduction activity (Fig. 9a), while TeO_3_^2−^ was reduced with the highest extent in the presence of NADH as electron donor (Fig. 9b). As a consequence of SeO_3_^2−^ and TeO_3_^2−^ reduction mediated by the cytoplasmic fraction, the ratio of NAD^+^/NADH increased in the case of TeO_3_^2−^ changing from 8.1 ± 0.4 to 17.7 ± 0.7 after 24-h incubation, while in the presence of SeO_3_^2−^ NAD^+^/NADH was comparable to that of the control (Fig. 9c).

**Fig. 10:**
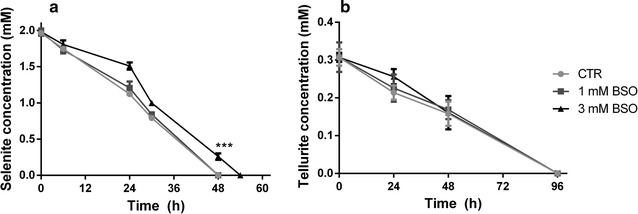
SeO_3_^2−^ (**a**) and TeO_3_^2−^ (**b**) reduction in presence of BSO. The effect of two different concentrations of BSO was tested (1 and 3 mM)

**Fig. 11:**
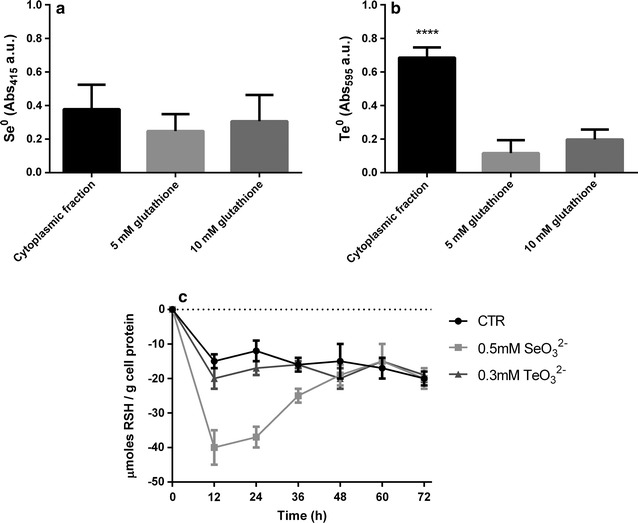
Comparison between cytoplasmic reduction and glutathione reduction of SeO_3_^2−^ (**a**) and TeO_3_^2−^ (**b**) and measurement of reduced thiols in cells exposed to SeO_3_^2−^ and TeO_3_^2−^ (**c**). The concentrations of glutathione used in **a**, **b** were 5 and 10 mM

The BSO effect on either SeO_3_^2−^ or TeO_3_^2−^ bioreduction was also evaluated (Fig. 10), revealing a 6-h delay of SeO_3_^2−^ bioreduction at the highest BSO concentration tested (3 mM) (Fig. 10a), while the presence of BSO did not affect TeO_3_^2−^ bioconversion (Fig. 10b), thus indicating that GSH seems to be not involved in this process. To support this hypothesis, we further compared the in vitro SeO_3_^2−^ and/or TeO_3_^2−^ reduction performed by L-GSH with the cytoplasmic fraction of *Ochrobactrum* sp. MPV1 (Fig. 11a, b). Indeed, both the cytoplasmic fraction and the solution containing 10 mM L-GSH comparably reduced SeO_3_^2−^ (Fig. 11a), while TeO_3_^2−^ reduction occurred with a minor extent in the presence of GSH molecules as compared to the cytoplasmic fraction (Fig. 11b). Moreover, RSH contents were measured after exposure of *Ochrobactrum* sp. MPV1 cells to 0.5 mM SeO_3_^2−^ and 0.3 mM TeO_3_^2−^ in comparison with non-exposed cells (Fig. 11c). As a result, MPV1 cells exposed to 0.5 mM SeO_3_^2−^ were featured by a loss of reduced thiols (− 40.3 ± 5.1 µmol RSH/g cell protein) as compared to those non-exposed (− 15.1 ± 2.0 µmol RSH/g cell protein) after 12-h incubation. On the opposite, MPV1 0.3 mM TeO_3_^2−^-exposed cells showed a thiols content (− 20.0 ± 3.3 µmol RSH/g cell protein) similarly to those non-exposed (Fig. 11c).

### FT-IR analysis on biogenic SeNPs and TeNPs

**Fig. 12:**
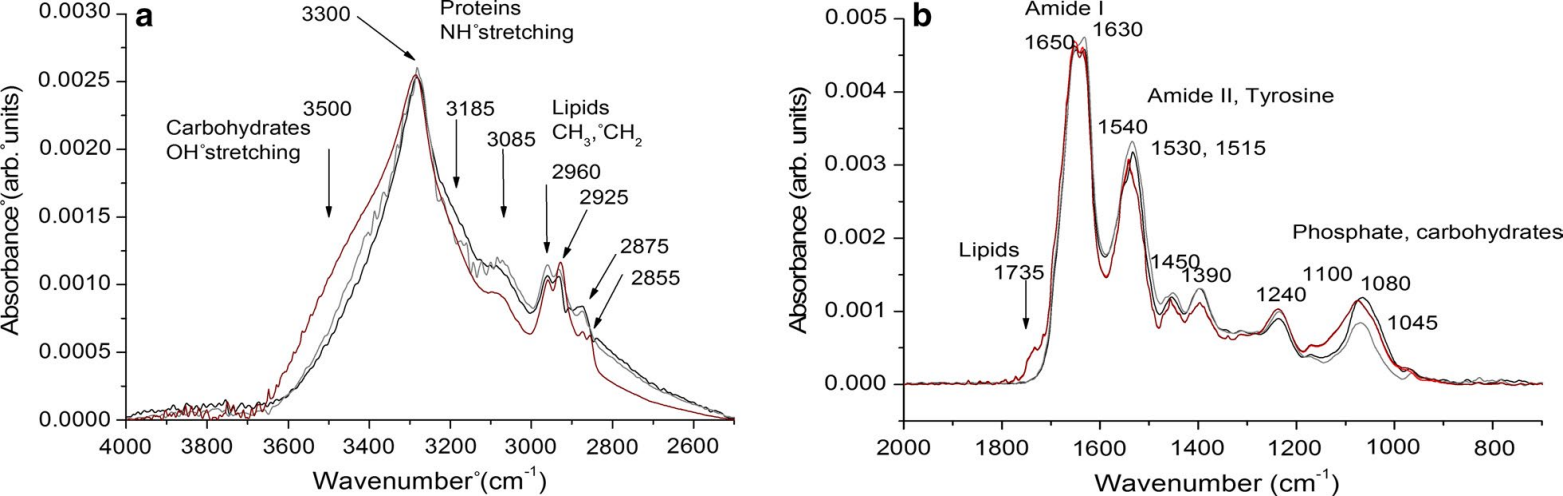
FTIR spectra of biogenic SeNPs and TeNPs extracted from *Ochrobactrum* sp. MPV1. Both SeNPs and TeNPs were extracted after 24 (T1) and 48-h (T2). Spectra were acquired in the 4000–2500 cm^−1^ (**a**) and 2000–700 cm^−1^ (**b**) infrared regions. SeNPs-T1 in dark red, SeNPs-T2 in light red, TeNPs-T1 in black, TeNPs-T2 in grey. SeNPs-T1 and SeNPs-T2 spectra are overlapped

Both biogenic SeNPs and TeNPs synthesized by *Ochrobactrum* sp. MPV1 after 24 (T1) and 48-h (T2) of growth were analyzed by FT-IR spectroscopy in the mid-infrared. Typical single point absorption spectra acquired on a 50 µm × 50 µm sample area are shown in Fig. 12a (4000–2400 cm^−1^ range) and Fig. 12b (1800–700 cm^−1^ range).

Main absorption bands related to the presence of functional chemical groups assigned to carbohydrates, proteins and lipids are indicated in Fig. 12 and described in Table 1.

**Table 1:** Main absorption bands and assignments related to biogenic SeNPs and TeNPs produced by *Ochrobactrum* sp. MPV1

Absorption bands (cm^−1^)	Main assignments
~ 3500	OH stretching in carbohydrates
~ 3300	NH stretching in proteins and peptides
~ 3185	Amide II overtone
~ 3085	NH stretching in proteins and peptides
~ 2960	CH_3_ asymmetric stretching in lipids
~ 2925	CH_2_ asymmetric stretching in lipids
~ 2875	CH_3_ symmetric stretching in lipids
~ 2855	CH_2_ symmetric stretching in lipids
~ 1735	C=O stretching in aliphatic polyesters
~ 1650/1630	Amide I
~ 1540/1530	Amide II
~ 1515	Tyrosine
~ 1450	CH_2_/CH_3_ bending vibrations in lipids and proteins
~ 1390	C=O of COO^−^ symmetric stretching in proteins
~ 1240	PO_2_ asymmetric stretching in phospholipids
~ 1100	Glycosidic linkage vibrations
~ 1080	PO_2_ symmetric stretching in phospholipids
~ 1045	C–O stretching modes in carbohydrates

Assignments are based on the literature [44–46]

To better understand the possible difference between the nanoparticles analyzed, Principal Component Analysis (PCA) was performed.

**Fig. 13:**
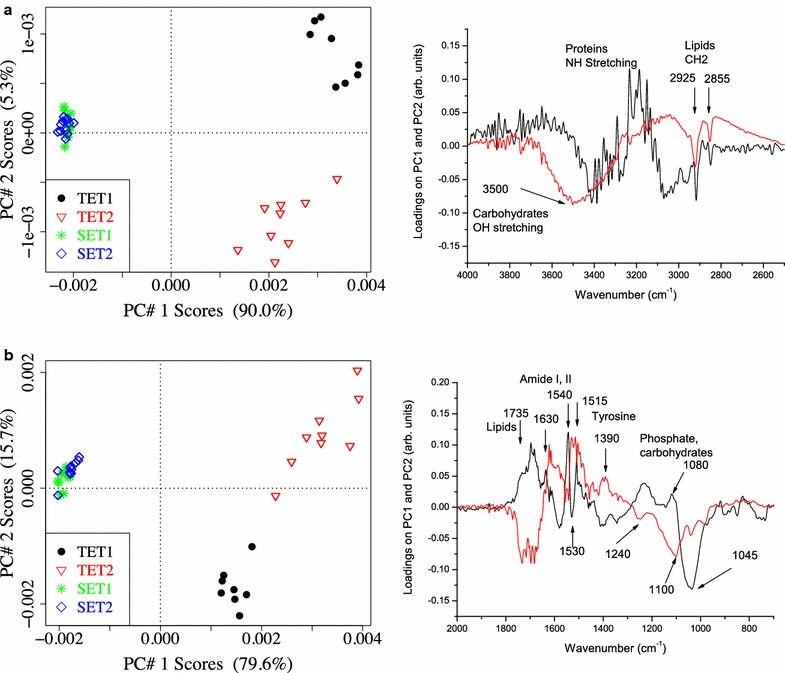
Principal Component Analysis on the FTIR spectra of SeNPs and TeNPs extracted from *Ochrobactrum* sp. MPV1. **a** PC1/PC2 score–score plot and loading plot for spectra acquired in the 4000–2500 cm^−1^ infrared region. Loadings of PC1 red line, loadings of PC2 black line. **b** PC1/PC2 score–score plot and loading for spectra acquired in the 2000–700 cm^−1^ infrared region. Loadings of PC1 red line, loadings of PC2 black line

PCA allows a graphical visualization of the projections of the spectra onto the PCs through ‘score–score plots’ that display the differences among the spectra as described by the first few PCs, which retain most of the original information measured by the percentage of captured variance. The loadings of each PC allow the explanation of these differences in terms of the relative intensities of the absorption bands: the spectral channels having positive (negative) loadings are more intense in the spectra with positive (negative) scores. In the present analysis, the first two PCs always best described the spread of the data. The PC1–PC2 score–score plots and the corresponding loadings are shown in Fig. 13.

The layers surrounding SeNPs and TeNPs show quite a different biochemical composition (PC1 scores). Moreover, SeNPs spectra at both incubation times are very similar, while spectra of TeNPs extracted after 24 and 48-h can be well distinguished (PC2 scores).

As a whole (PC1 loadings, red lines), SeNPs show higher intensities of the bands related to lipids (at 2925 cm^−1^, CH_2_ asymmetric stretching; at 1735 cm^−1^, C=O stretching in aliphatic polyesters) and phosphate (at 1240 and 1080 cm^−1^, PO_2_ asymmetric and symmetric stretching) and of the bands related to carbohydrates (3500 cm^−1^, OH stretching; and 1100 cm^−1^, glycosidic linkage). The higher intensity of CH_2_ stretching vibrations can be due to an increased length of the lipid chains to which the ratio CH_2_/CH_3_ is directly related.

On the contrary (PC1 loadings, red lines), TeNPs show higher intensities of the bands related to proteins, namely the amide II band at 1540 cm^−1^, the tyrosine band at 1515 cm^−1^ and the C=O stretching band at 1390 cm^−1^. Interestingly (PC2 loadings, black lines), in TeNPs after 48-h of incubation absorption bands related to proteins (1630, 1540, 1515 cm^−1^), lipids (2925, 2855 and 1735 cm^−1^) and phosphate (1240 and 1080 cm^−1^) all have higher intensities than after 24-h, while the band at 1045 cm^−1^, related to carbohydrates, neatly decreases.

## Discussion

In the present study *Ochrobactrum* sp. MPV1, previously isolated from arsenopyrite ashes dumped near a formerly industrial site operating in Italy for the production of sulfuric acid by the pyrite roasting process, was investigated for the ability to tolerate and reduce SeO_3_^2−^ and TeO_3_^2−^ in aerobic conditions, focusing on the possible mechanism/s responsible for the conversion of these toxic oxyanions to the zero-valent Se and Te-nanostructures.

The genus *Ochrobactrum* has been described the first time by Holmes et al. [47]. Several strains belonging to this genus have shown elevated levels of resistance towards heavy metals [48] and metalloids [49]. In addition, the role of *Ochrobactrum* species in promoting plant growth in polluted environments has been recently reported [50]. The tolerance towards SeO_3_^2−^ of *Ochrobactrum* sp. MPV1 exceeded the level of resistance exhibited by the most part of isolates reported in literature, which is between 5–20 mM Na_2_SeO_3_ [51], although bacterial strains with a SeO_3_^2−^ tolerance exceeding 100 mM have been described [52–54]. Conversely, the MPV1 strain can tolerate lower concentrations of TeO_3_^2−^, which is consistent with previous studies indicating the higher TeO_3_^2−^ toxicity as compared to other metals and metalloids of environmental and public health concern [55]. Indeed, TeO_3_^2−^ is known to be toxic to most bacteria at concentrations as low as 1 µg mL^−1^ [13]. However, tolerant strains have been isolated and studied, such as *Stenotrophomonas maltophilia* Sm777 [56] or *Paenibacillus* sp. TeW [57], with a level of TeO_3_^2−^ resistance similar to *Ochrobactrum* sp. MPV1. Additionally, different strains exhibited even higher level of resistance to TeO_3_^2−^, such as *Salinicoccus* sp. strain QW6 [52] and *Rhodococcus aetherivorans* BCP1 [58].

MPV1 strain was able to completely convert 0.5 and 2 mM SeO_3_^2−^ within 30 and 48-h respectively (Fig. 3a, b), with efficiency comparable to *Bacillus mycoides* SeITE01 [59]. Other bacterial strains have been tested for SeO_3_^2−^ bioreduction under aerobic conditions: for instance, *S. maltophilia* SeITE02 [25] was able to bioreduce 0.5 mM SeO_3_^2−^ within 48-h, while the complete reduction of 2 mM SeO_3_^2−^ was not observed even after 140-h of incubation. Similarly, *Burkholderia fungorum* DBT1 and 95 were able to completely bioreduce 0.5 mM SeO_3_^2−^ within 96-h, while the bioconversion of 2 mM SeO_3_^2−^ was not completed during the same timeframe [60]. It is noteworthy to mention that *Pseudomonas moraviensis* subsp. stanleyae efficiently removed 10 mM SeO_3_^2−^ in 48-h [61].

Although the literature reports on remarkable TeO_3_^2−^ resistance and bioreduction potential mediated by aerobic bacterial phototrophs [62], it is worth noting that a high extent of TeO_3_^2−^ bioreduction was described for other strains belonging to the *Ochrobactrum* genus, namely *Ochrobactrum anthropi* TI-2 and TI-3, able to completely reduce 1 mM of TeO_3_^2−^ within 30-h [49]. In this regard, MPV1 strain resulted capable of bioreducing 0.3 and 0.5 mM of TeO_3_^2−^ within 72 and 120-h of growth, as in the case of *Salinicoccus* sp. strain QW6 [52], while the bioconversion of the highest TeO_3_^2−^ concentration tested (1 mM) resulted to be ca. 70% during 120-h growth (Fig. 4b), suggesting a toxic effect of this TeO_3_^2−^ concentration as highlighted by the presence of a 48-h lag phase (Fig. 4a). Therefore, the lag phase featuring MPV1 TeO_3_^2−^-grown cells suggested that the bioprocessing of this oxyanion is an inducible process as compared to the one of SeO_3_^2−^. Moreover, pre-induced cells with sub-lethal concentrations of either SeO_3_^2−^ or TeO_3_^2−^ displayed unchanged SeO_3_^2−^ bioreduction extents (Fig. 5a). Similarly, TeO_3_^2−^ pre-induction did not affect the bioconversion of this oxyanion, while an incomplete TeO_3_^2−^ bioreduction was detected in the case of SeO_3_^2−^ pre-induced cells (Fig. 5b), further suggesting that MPV1 may exploit different mechanisms to bioprocess diverse chalcogen-oxyanions. To this aim, in vitro and in vivo assays were performed to assess the mechanism behind SeO_3_^2−^ and/or TeO_3_^2−^ transformation processes. Thus, among the electron donors tested, SeO_3_^2−^ reduction was most efficient upon addition of NADPH in the reaction mixture as compared to NADH and reduced ascorbate (Fig. 9a). Indeed, NAD^+^/NADH ratio was comparable to the one of MPV1 cells non-exposed to SeO_3_^2−^ (Fig. 9c). Further, since NADPH have been described as the preferential electron donor utilized by GSH reductases [41], these enzymes might play a key role in SeO_3_^2−^ reduction process, as indicated also by the delayed SeO_3_^2−^ reduction occurred upon addition of 3 mM BSO (inhibitor of GSH biosynthesis) to MPV1 cultures (Fig. 10a). Additionally, the cytoplasmic fraction isolated from MPV1 cells exhibited comparable reduction extent to 10 mM L-GSH (Fig. 11a), being these results consistent with those reported by Kessi and Hanselmann [41] in the case of *Rhodospirillum rubrum* [41]. The involvement of GSHs in SeO_3_^2−^ bioreduction was further supported by a strong decreased RSH content observed in MPV1 cells exposed to this oxyanion, as a consequence of SeO_3_^2−^ bioconversion (Fig. 11c). All these findings strengthen the hypothesis that GSH is involved in the SeO_3_^2−^ bioreduction process exploited by *Ochrobactrum* sp. MPV1.

The biochemical mechanisms responsible for TeO_3_^2−^ bioreduction to Te^0^ has yet to be elucidated. However, several studies reported that NADH-dependent enzymes, such as catalase [63], dihydrolipoyl dehydrogenase [64], α-ketoglutarate, isocitrate dehydrogenase [65], and NADH-II dehydrogenase [66] might mediate TeO_3_^2−^ bioconversion. More recently, two different periplasmic TeO_3_^2−^ and SeO_3_^2−^ reductases using glutamate as electron donor were isolated from the strain ER-Te-48, which is phylogenetically related to *Shewanella frigidimarina* [67]. In our study, NADH other than NADPH and reduced ascorbate resulted to be the most efficient electron donor mediating TeO_3_^2−^ reduction in the cytoplasmic fraction recovered from MPV1 cells (Fig. 9b), as also indicated by the depletion of NADH as well as the increased NAD^+^/NADH ratio observed after 24-h exposure to TeO_3_^2−^ (Fig. 9c). Moreover, since (i) BSO addition did not affect TeO_3_^2−^ bioreduction (Fig. 10b), (ii) either 5 or 10 mM L-GSH did not display a comparable reduction extent with the one of the cytoplasmic fraction (Fig. 11b), and (iii) RSH content of TeO_3_^2−^-exposed cells was comparable to that of non-exposed ones (Fig. 11c), it is reasonable to suggest that a NADH-dependent enzyme may be responsible for TeO_3_^2−^ bioconversion in *Ochrobactrum* sp. MPV1.

Consequently to either SeO_3_^2−^ or TeO_3_^2−^ bioconversion, *Ochrobactrum* sp. MPV1 was able to generate Se- and Te-nanomaterials, as depicted by transmission electron micrographs (Fig. 6). Biogenic nanostructures were intracellularly produced, as also highlighted by the absence of nanomaterials outside MPV1 cells (Fig. 7a, b), as well as the detection of Se and Te specific absorption peaks revealed by EDAX analyses on cellular samples (Fig. 7c, d). Particularly, TEM images showed the presence of Se- and Te-nanostructures featured by different morphologies, i.e., NPs (Se and Te) and short needle-like NRs (Te) (Fig. 6f1). These observations are in line with previous studies regarding Se- and Te-nanomaterial production by other bacterial strains (e.g., *Bacillus beveridgei* MLTeJB and *Shewanella oneidensis* MR-1), which showed to generate chalcogen nanostructures featured by different morphologies [68, 69]. Moreover, SeO_3_^2−^-grown bacteria were described to produce Se-nanomaterials mostly in the form of NPs [70], while TeNRs are usually generated as a result of microbial growth in the presence of TeO_3_^2−^ [58, 71], due to the intrinsic crystalline nature of Te^0^ atoms [72].

Since previous studies concerning the characterization of biogenic nanomaterials indicated the presence of an organic layer playing a key role in their synthesis and stabilization [73], in this study FT-IR spectroscopy was performed for the first time to evaluate the nature of the organic coating of Se- and TeNPs produced by the MPV1 strain (Figs. 12, 13). FT-IR spectra highlighted mostly the presence of bands related to phosphate groups and lipids for SeNPs, while proteins resulted to be the main component of the organic layer in the case of TeNPs (Fig. 12), suggesting a different composition in the coating of biogenic chalcogen-NPs. Similarly to our observations, FT-IR analysis performed on biogenic SeNPs produced by *S. maltophilia* SeITE02 revealed the presence of carbohydrates, lipids and proteins [25], while TeNPs synthesized by *Rhodobacter capsulatus* B100 showed peaks related to proteins and carbohydrates [24]. Moreover, SeNPs extracted after 24 or 48-h from MVP1 cells did not show strong differences in the band intensities detected by FT-IR spectroscopy (Fig. 13). Conversely, a drastic increase in the bands related to proteins, phosphate groups and lipids was observed for TeNPs recovered after 48-h from TeO_3_^2−^-grown cells, indicating that a maturation process of these nanomaterials could take place during bacterial growth (Fig. 13). Nevertheless, deeper investigations are needed to elucidate the macromolecular composition of the organic coating surrounding both SeNPs and TeNPs biosynthesized by MPV1 strain.

In conclusion, the results obtained in the present study suggested that *Ochrobactrum* sp. MPV1 most likely exploited two different mechanisms to bioprocess SeO_3_^2−^ and TeO_3_^2−^, which might be mediated by GSHs and intracellular NADH-dependent oxidoreductases, respectively. Further, the characterization of the organic layers surrounding biogenic Se- and TeNPs revealed a diverse macromolecular composition, emphasizing the differences on which the two oxyanions bioprocessing mechanisms are based. Eventually, the present study demonstrated the possibility to use *Ochrobactrum* sp. MPV1 as a suitable cell factory to bioconvert toxic SeO_3_^2−^ and TeO_3_^2−^ and finally produce biogenic NPs.
